# Unidirectional Far‐Field Radiation of Surface Waves by On‐Chip Achromatic Metasurfaces

**DOI:** 10.1002/nap2.70064

**Published:** 2026-04-10

**Authors:** Haodong Li, Shaojie Ma, Lei Zhou, Shulin Sun

**Affiliations:** ^1^ Department of Physics State Key Laboratory of Surface Physics and Key Laboratory of Micro and Nano Photonic Structures (Ministry of Education) Fudan University Shanghai China; ^2^ Department of Optical Science and Engineering Shanghai Engineering Research Center of Ultra‐Precision Optical Manufacturing College of Future Information Technology Fudan University Shanghai China; ^3^ Shanghai Key Laboratory of Metasurfaces for Light Manipulation Shanghai China; ^4^ Key Laboratory of Micro and Nano Photonic Structures (Ministry of Education) Fudan University Shanghai China

**Keywords:** achromatic metasurfaces, far‐field radiation, Pancharatnam–Berry phase, resonant phase, surface wave

## Abstract

Converting surface waves to unidirectional far‐field beams is crucial for information readout in on‐chip photonic applications, but conventional grating‐like couplers suffer from low‐efficiency issue due to multi‐mode generation. Although recently proposed metasurfaces have addressed this challenge, meta‐couplers realized so far exhibit inevitable frequency dispersions, restricting their practical applications. Here, we propose a scheme to design on‐chip achromatic metasurfaces that can decouple SWs within a continuous frequency band to far‐field beams traveling along the same direction. Our metadevices, composed of composite meta‐atoms exhibiting pre‐designed reflection phases with tailored frequency dispersion dictated by both resonant and Pancharatnam–Berry phase mechanisms, can compensate the desired frequency‐dependent wavevectors for SWs at different frequencies, thereby generating unidirectional far‐field beams. As a proof of concept, we design and fabricate two on‐chip achromatic metasurfaces working in the microwave regime and experimentally demonstrate that they can achieve in‐plane and out‐of‐plane unidirectional far‐field decoupling of SWs, exhibiting relative frequency bandwidths of 22.22% and 37.04%, respectively. Both microwave experiments and full‐wave simulations successfully confirm our theoretical predictions. These findings pave the way for on‐chip photonic manipulations and related applications, including wireless communication, signal processing, high‐sensitivity sensing, and so on.

## Graphical abstract

Converting surface waves to unidirectional far‐field beams is essential for on‐chip photonics, but traditional couplers often suffer from inefficiency and frequency dispersion. This work experimentally demonstrates the concept of on‐chip achromatic metasurfaces, which can efficiently decouple broadband surface waves into unidirectional far‐field beams. This finding paves the way for integrated photonic applications, including wireless communication, signal processing, and high‐sensitivity sensing.

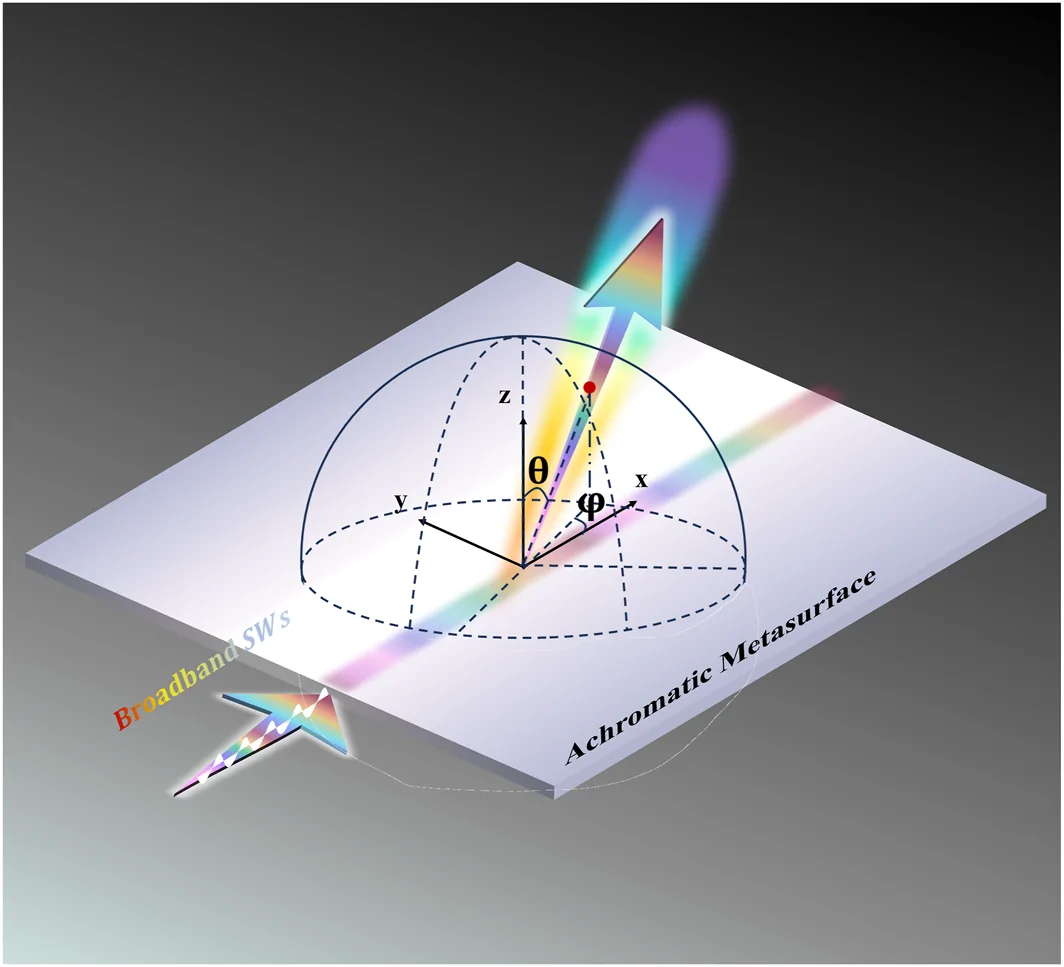

**Abbreviations**
CP: circular polarization
DSRRs: double split‐ring resonators
FF: far‐field
MIM: metal‐insulator‐metal
MS: metasurface
NF: near‐field
PB: Pancharatnam–Berry
PW: propagation wave
RCP: right‐handed circular polarization
SW: surface wave

## 1 Introduction

On‐chip integrated devices, employing surface waves (SWs) as information carriers, offer an ultra‐compact platform to transport and manipulate electromagnetic (EM) waves [1, 2]. These planar devices can yield many applications such as optical sensing, high‐speed computing, wireless communication, and so on. To extract information from on‐chip systems, grating‐like couplers are widely utilized to decouple SWs to far‐field (FF) propagation waves (PWs) through inverse wavevector compensation [3, 4]. However, Bragg devices typically face the low‐efficiency challenge caused by multi‐mode generation. Moreover, due to the dispersive nature of PWs, such devices may decouple SWs at different frequencies to PWs traveling along distinct directions [3, 4], which severely degrades the working performances of our devices as information detectors. Therefore, it is highly desired to achieve achromatic decoupling of SWs within a continuous band to the same FF channel.

Metasurfaces (MSs), planar EM devices composed of sub‐wavelength microstructures exhibiting carefully designed EM responses, offer us powerful capabilities to flexibly modulate the wavefronts of EM waves both in FF and near‐field (NF) [5, 6, 7, 8]. Different from grating couplers replying on Bragg‐scattering mechanisms, MSs can locally manipulate phase retardations of EM waves at deep‐subwavelength scales, yielding fascinating wave‐control effects based on Huygens' principle, such as anomalous reflection/refraction [5, 9, 10, 11], flat EM focusing [12, 13, 14, 15], meta‐holography [16, 17, 18, 19], etc. Furthermore, researchers also demonstrated that MSs are able to couple FF PWs into NF SWs exhibiting predefined wavefronts, paving a new way to efficiently excite on‐chip optical devices [6, 20, 21, 22, 23]. On the other hand, MSs were also designed based on a specific phase mechanism, that can decouple SWs supported by on‐chip devices to scalar or vectorial PW beams with arbitrary wavefronts, achieving novel effects such as directional radiation [24, 25], light focusing [26, 27, 28], and special beam generation [29, 30, 31, 32]. By integrating two distinct phase‐control mechanisms (e.g., geometric phase and detour phase), people have realized integrated MSs to achieve multi‐channel wave controls modulated by wavelength or polarization, such as multiplexed holography for augmented reality [33, 34, 35, 36], multifunctional focusing [37], and so on. However, these MSs are usually designed at a single frequency, which inevitably suffer from the issue of chromatic aberrations.

In a parallel line, various achromatic MSs have been demonstrated, based on carefully optimized meta‐atoms exhibiting individually designed dispersive phase responses under excitation of PWs [38, 39, 40]. Early proposed achromatic metadevices were designed for several discrete frequencies through exhaustively searching available ranges of multiple geometric parameters of constitutional meta‐atoms. However, such a design strategy is not only time‐consuming but also unfavorable for broadband applications. Alternatively, continuous‐band achromatic MSs were further developed for achieving wavefront controls such as anomalous beam deflection [39], beam focusing [41], airy beam generation [42], cloak [43], and so on. While designing these MSs, one needs to sort out a series of meta‐atoms, which not only exhibit the target phase profile at a single frequency but also the desired frequency‐variation slopes within the entire frequency band. However, all these efforts were devoted to achromatic MSs under PW excitations. Recently, several groups have realized “achromatic” metagratings that can decouple the waveguide SW modes at red, green, and blue (RGB) wavelengths to FF beams traveling along the same direction, through leveraging different diffraction orders of RGB lights [44, 45, 46]. Unfortunately, these devices still exhibit chromatic aberrations for continuous‐band light and may encounter the low efficiency issue due to multiple mode generations.

In this work, we propose a scheme to design achromatic on‐chip metadevices that can decouple SWs across a continuous broad band to the unidirectional FF radiation channel, as shown in Figure 1. Different from metagratings, our devices, governed by Huygens' principle, are composed of a series of subwavelength meta‐atoms exhibiting individually optimized phases and frequency dispersions based on both Pancharatnam–Berry (PB) and resonant phase mechanisms. As a proof of concept, we design and fabricate a microwave MS and experimentally demonstrate that it can decouple SWs within [8 GHz, 10 GHz] to the same in‐plane FF radiation channel carrying a specific circular polarization (CP). In addition, we further experimentally demonstrate another SW‐driven achromatic metadevice, consisting of more complicated microstructures, that can realize the out‐of‐plane unidirectional emission of SWs within a broader band of [8.8 GHz, 12.8 GHz]. Full wave simulations are in nice agreement with NF mapping measurements, which collectively verify our theoretical proposal. The achromatic on‐chip metasurfaces developed in this work address the fundamental issues of multimode generation, discrete working wavelengths, and low efficiency existing in previous achromatic metagratings [44, 45, 46].

**FIGURE 1:**
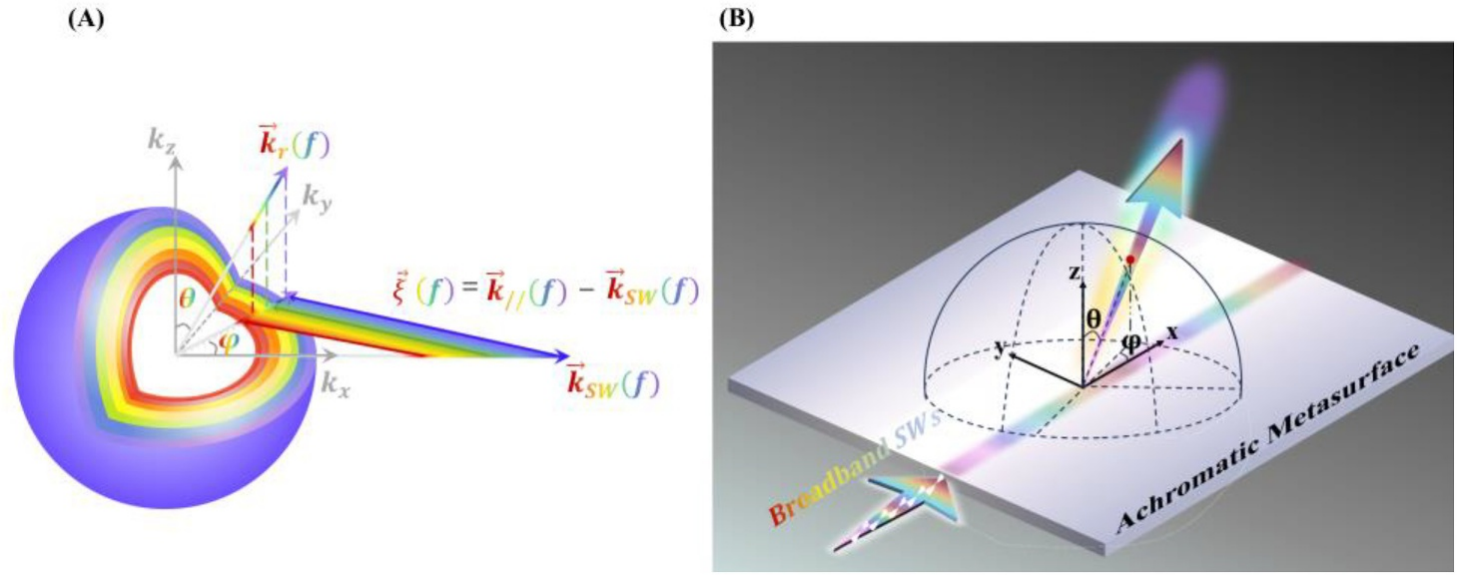
(A) Schematic of parallel wavevector conversation law satisfied by unidirectional radiation field generated by SW excited on‐chip achromatic MS; (B) schematic of unidirectional FF radiation by the on‐chip MS excited by SW within a continuous band.

## 2 Results

### 2.1 Generic Design Strategy

In this section, we introduce our scheme to design the broadband achromatic on‐chip MS. To achieve the unidirectional FF radiation of impinging SW across a continuous frequency band $\left[f_{\min},f_{\max}\right]$, we need to design an MS that can introduce frequency‐dependent phase gradient $\vec{\xi }\left(f\right)$ to the SW flowing on the device. In this process, the parallel wavevector ${\vec{k}}_{//}\left(f\right)$ of the unidirectional PWs scattered by the SW‐excited MS must follow the conservation law (see Figure 1A):

(1) $${\vec{k}}_{//}\left(f\right)={\vec{k}}_{\text{SW}}^{in}\left(f\right)+\vec{\xi }\left(f\right).$$

where ${\vec{k}}_{\text{SW}}^{in}$ is the wavevector of incident SWs and f is the frequency. The predefined direction of the broadband radiation beams emitted by the achromatic MS can be described by $\theta_{0},\phi_{0}$:

(2) $$k_{x}^{\text{rad}}\left(f\right)=k_{0}\left(f\right)\sin\;\theta_{0}\;\cos\;\phi_{0}$$

(3) $$k_{y}^{rad}\left(f\right)=k_{0}\left(f\right)\sin\;\theta_{0}\;\sin\;\phi_{0}$$

According to Equations ((1), (2), (3)), we can easily derive the required phase gradient factors $\xi_{x}\left(f\right)$ and $\xi_{y}\left(f\right)$ of the SW driven (e.g., flowing along the +*x* direction) achromatic MS:

(4) $$\xi_{x}\left(f\right)=-k_{\text{SW}}\left(f\right)+k_{0}\left(f\right)\sin\;\theta_{0}\;\cos\;\phi_{0}$$

(5) $$\xi_{y}\left(f\right)=k_{0}\left(f\right)\sin\;\theta_{0}\;\sin\;\phi_{0}$$

Based on Equations (4) and (5), we can easily derive the frequency‐dependent phase profile encoded inside the desired achromatic MS:

(6) $$\Phi \left(f,x,y\right)=\left(-k_{\text{SW}}\left(f\right)+\frac{2\pi }{c_{0}}f*\sin\;\theta_{0}\;\cos\;\phi_{0}\right)\cdot \left(x-x_{0}\right)+\frac{2\pi }{c_{0}}f*\sin\;\theta_{0}\;\sin\;\phi_{0}\cdot \left(y-y_{0}\right)\;f\in \left[f_{\min},f_{\max}\right]$$

where $c_{0}$ is the speed of electromagnetic (EM) waves in vacuum and (x,y) denotes the spatial location of meta‐atoms on the MS. Equation (6) implies that phase retardations provided by our achromatic MS should compensate the propagation phases accumulated along the optical path of SWs flowing on the device and the radiation PW beam in free space, thereby fulfilling the constructive condition for the unidirectional radiation beams across a broad frequency band. Considering that the eigen wavevector of SW almost satisfies the nearly linear frequency dispersion, the phase retardation Φ of the meta‐atoms at different positions of (x,y) should vary linearly against the frequency but with distinct slopes. Meanwhile, at a fixed frequency (e.g., $f_{\min}$), $\Phi \left(f_{\min},x,y\right)$ needs to satisfy a specific spatial distribution.

Guided by the above theoretical analysis, we formulate the strategy to design the achromatic on‐chip MSs, which are constructed by the composite meta‐atoms encoded with two different phase modulation mechanisms:

(7) $$\Phi \left(f,x,y\right)=\phi^{r\mathrm{e}s}\left(f,x,y\right)+\phi^{\text{PB}}\left(x,y\right)$$

where $\phi^{r\mathrm{e}s}\left(f,x,y\right)$ and $\phi^{\text{PB}}\left(x,y\right)$ denote the frequency‐dependent resonant phase and dispersionless PB phase, respectively. And these two terms can be flexibly modulated via varying two individual degrees of freedom, that is, the structural geometries and orientation angles of the meta‐atoms, respectively. At the position of (x,y), we can design the meta‐atoms exhibiting a linearly dispersive resonant phase with desired slope via elaborately optimizing the numbers and frequencies of their resonant modes:

(8) $$\frac{d\phi^{r\mathrm{e}s}}{df}=-\frac{dk_{SW}}{df}+\frac{2\pi }{c_{0}}\sin\;\theta_{0}\;\cos\;\phi_{0}\left(x-x_{0}\right)+\frac{2\pi }{c_{0}}\sin\;\theta_{0}\;\sin\;\phi_{0}\left(y-y_{0}\right)$$

although the obtained meta‐atoms satisfy the desired phase slope determined by Equation (8), they usually cannot satisfy another constraint, that is, the phase profile $\Phi \left(f_{\min},x,y\right)$ at the initial frequency. To address this issue, we can further introduce the frequency‐independent PB phase via precisely tuning the orientation angles of meta‐atoms located at (x,y):

(9) $$\phi^{\text{PB}}\left(x,y\right)=\Phi \left(f_{\min},x,y\right)-\phi^{r\mathrm{e}s}\left(f_{\min},x,y\right)$$

Until now, we have finished the whole design process of the desired achromatic on‐chip MS for achieving unidirectional FF radiation of SW within the target frequency band $\left[f_{\min},f_{\max}\right]$. It should be noted that the generated achromatic beam is spin‐polarized since the geometric phase is introduced into the design of MS. Considering that the spin‐dependent PB phase is determined by $\phi^{PB}=\sigma 2\Omega$, where σ=±1 describes the chirality of radiation beams (σ = −1: left‐handed circular polarization (LCP); σ = +1: right‐handed circular polarization (RCP)) and Ω is the orientation angle of the meta‐atom. Therefore, we can freely select one particular chirality for the radiation beams via selecting orientation sequence of composite meta‐atoms of the MS.

### 2.2 In‐Plane Unidirectional Radiation of SW Across a Broad Frequency Band

Without loss of generality, we choose the microwave regime to validate our concept. We first design the building blocks required to create the SW‐driven achromatic MS, including the meta‐atoms and plasmonic metal for supporting SW. The meta‐atom, designed in metal‐insulator‐metal (MIM) configuration, consists of double split rings and a patch resonator, a 2 mm‐thick FR4 spacer $\left(\varepsilon_{\text{FR}4}=4.3+0.005i\right)$, and a continuous metallic film, as schematically shown in Figure 2A. Generally, the reflection coefficient $r_{a}$ of the anomalous mode emitted by each meta‐atom can be described by the following form:

(10) $$r_{a}=\left|r_{a}\right|e^{i\phi_{a}}=\left|\frac{r_{uu}{-r}_{vv}}{2}\right|e^{i\left(\phi^{r\mathrm{e}s}+\phi^{\text{PB}}\right)}$$

(11) $$\phi^{\text{res}}=\arg\left(\frac{r_{uu}{-r}_{vv}}{2}\right)$$

(12) $$\phi^{\text{PB}}=\sigma 2\Omega$$

here, $r_{uu}$ and ${\;r}_{vv}$ denote the complex reflection coefficients of the meta‐atoms arranged in a periodic array illuminated by incident light linearly polarized along two principal axes (*u* and *v* axes), respectively. We can note that the anomalous mode of such meta‐atoms exhibits both resonant and PB phases, which are crucial to achieve the achromatic functionality.

We now elaborate on the process of designing a series of meta‐atoms to create the achromatic on‐chip MS for achieving unidirectional FF radiation of broadband SW. Initially, we engineer the geometric parameters of two coupled microstructures on the meta‐atoms (r,c,g and l,w) to achieve both the high anomalous reflection amplitude $\left|r_{a}\right|$ and desired reflection phase slopes $\frac{d\phi^{r\mathrm{e}s}}{df}$. In this step, our objective is to obtain the meta‐atoms behaving similar to half‐waveplates, thereby enhancing the intensity of desired anomalous radiation mode. Meanwhile, we engineer the dispersion behavior (i.e., resonant phase slope) of the meta‐atoms via optimizing the number and frequencies of the supported resonant modes.

Following this strategy, we establish a database of meta‐atoms for creating high‐performance on‐chip achromatic metadevices working within a continuous band. As examples, we introduce two representative meta‐atoms (meta‐atom I and meta‐atom II) to illustrate their designs and performances, as shown in Figure 2B,D. In our calculations, the meta‐atoms arranged in a square lattice are illuminated by normally incident CP light. Full wave simulations reveal that the amplitudes of anomalous reflection mode $\left|r_{a}\right|$ for both meta‐atoms are quite high within the target frequency band of 8–10 GHz, which is highly desired for creating the achromatic MS. In addition, Figure 2B shows that the meta‐atom I, characterized by two widely separated modes $\omega_{A}^{I}=6.64$ GHz, $\omega_{B}^{I}=11.85$ GHz with low quality factors, exhibits a linear resonant phase with a small slope across target band. In comparison, the meta‐atom II depicted in Figure 2D achieves a much larger phase slope due to the increase of the mode numbers: $\omega_{A}^{\text{II}}=7.40$ GHz, $\omega_{B}^{\text{II}}=9.03$ GHz, $\omega_{C}^{\text{II}}=14.66$ GHz. Leveraging this method, we have developed a large database of meta‐atoms that can provide a diverse range of phase‐dispersive responses (see more details in Section A of the Supporting Information S1).

**FIGURE 2:**
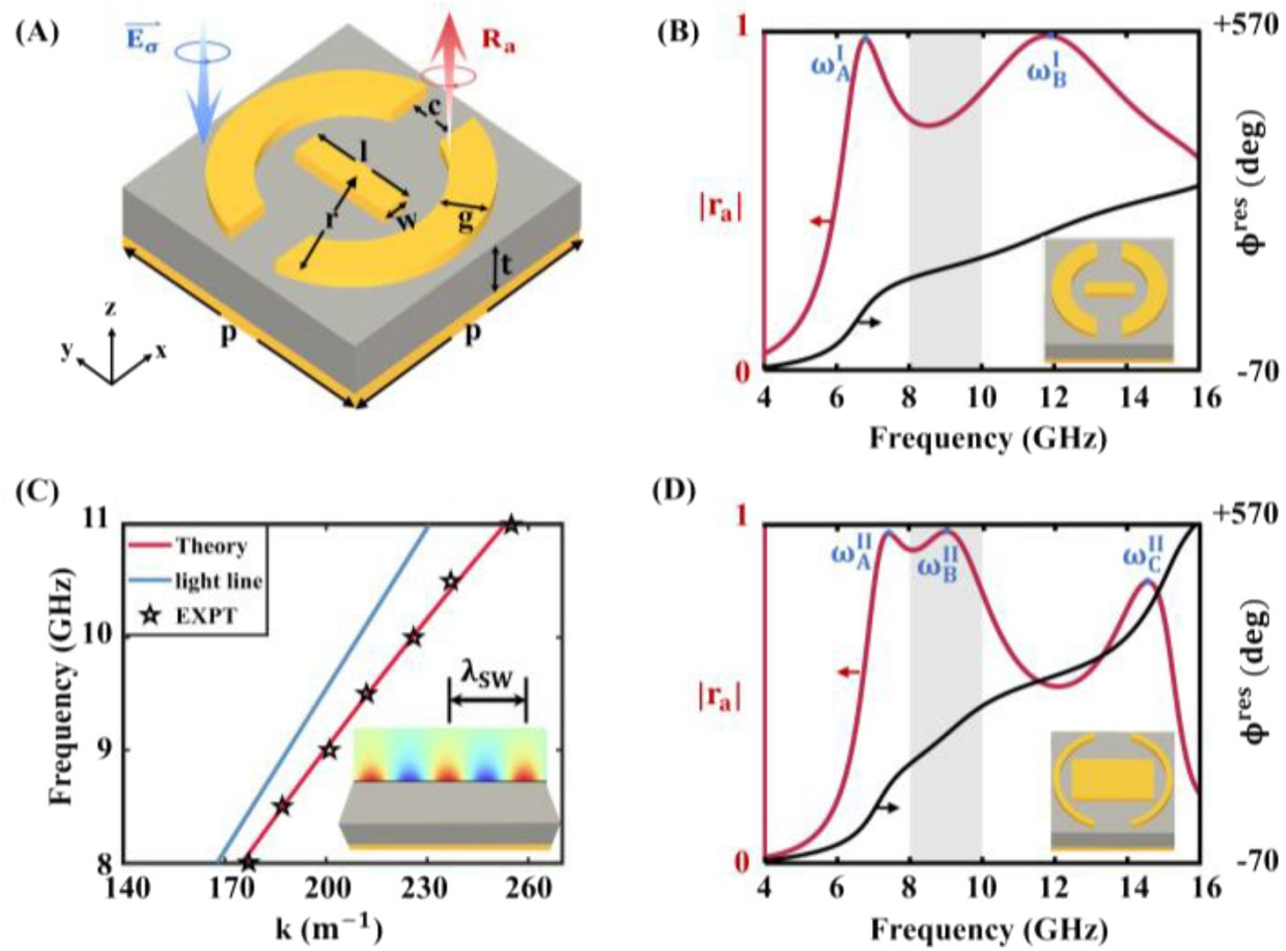
Characterization of representative meta‐atoms for creating achromatic MS for in‐plane unidirectional radiation. (A) Schematic of proposed meta‐atom composed of double split ring resonators (DSRRs) and a patch and the copper film that are separated by 2 mm thick FR‐4 dielectric substrate ($\varepsilon_{\text{FR}4}=4.3+0.005i$). Here, p= 10 mm, t= 2 mm. (B, D) Simulated spectra of resonant phase and anomalous reflection mode amplitude ($\left|r_{a}\right|$) for two representative meta‐atoms. These building blocks exhibit quite good linear resonant phase dispersion with different slopes across the target band of 8–10 GHz. The geometric parameters are listed as follows: r=3 mm, c=2 mm, g=1.5 mm and l=3.864 mm, w=1.035 mm for meta‐atom I (B), r=4.15 mm, c=4 mm, g=0.5 mm and l=6.322 mm, w=3.65 mm for meta‐atom II (D). (C) Dispersion relation of SW supported by the plasmonic metal shown in the inset, obtained by theoretical calculation (red line) and NF measurements (black pentagram).

We further design “plasmonic metal” to support eigen SWs (i.e., the spoof surface plasmon mode) in the microwave regime, where the metals are too conductive to support natural surface plasmon mode. The “plasmonic metal” is simply composed of a continuous metallic thin film and a dielectric spacer whose thickness is exactly the same as that of the previous meta‐atom (see inset of Figure 2C). Dispersion relation of the eigen SWs supported by such a “plasmonic metal,” primarily governed by the dielectric properties and geometric thickness of the spacer layer, is analytically calculated based on the transfer matrix method, which shows nice agreement with our experimental measurements based on NF scanning technique (see more details in Section B of the Supporting Information S1).

After obtaining all the building blocks, we design and fabricate the first SW‐driven achromatic MS to achieve in‐plane ($\phi_{1}^{0}=0{}^{\circ}$) unidirectional radiation effect across the target band of 8–10 GHz, as schematically shown in Figure 3A. According to the Equation (6), we find that such a metadevice should exhibit the following phase distribution:

(13) $$\Phi \left(f,x\right)=\left[-k_{\text{SW}}\left(f\right)+\frac{2\pi }{c_{0}}f*\sin\;\theta_{1}^{0}\right]\left(x-x_{0}\right)f\in \left[f_{\min},f_{\max}\right]$$

where $\theta_{1}^{0}=44.6{}^{\circ}$ is the pre‐defined dispersionless radiation angle within the band of 8–10 GHz. We initially select a series of appropriate meta‐atoms from the database exhibiting the correct phase‐variation slopes $\frac{\partial \Phi }{\partial f}{|}_{x}=\frac{d\phi^{r\mathrm{e}s}}{df}=\left(-\frac{dk_{\text{SW}}}{df}+\frac{2\pi }{c}\sin\;\theta_{1}^{0}\right)\left(x-x_{0}\right)$ located at different x positions. We next assign specific orientation angles for the different meta‐atoms to fulfill the required phase profile $\Phi \left(f_{\min},x\right)$ at the initial frequency $f_{\min}$ = 8 GHz according to Equations (9) and (12). Here, we choose RCP with σ=1 as polarization state of the radiation beams. Figure 3C depicts the simulated spatial phase distributions of the practically designed achromatic MS for in‐plane unidirectional radiation at several representative frequencies, showing good agreement with theoretically designed phase profiles.

**FIGURE 3:**
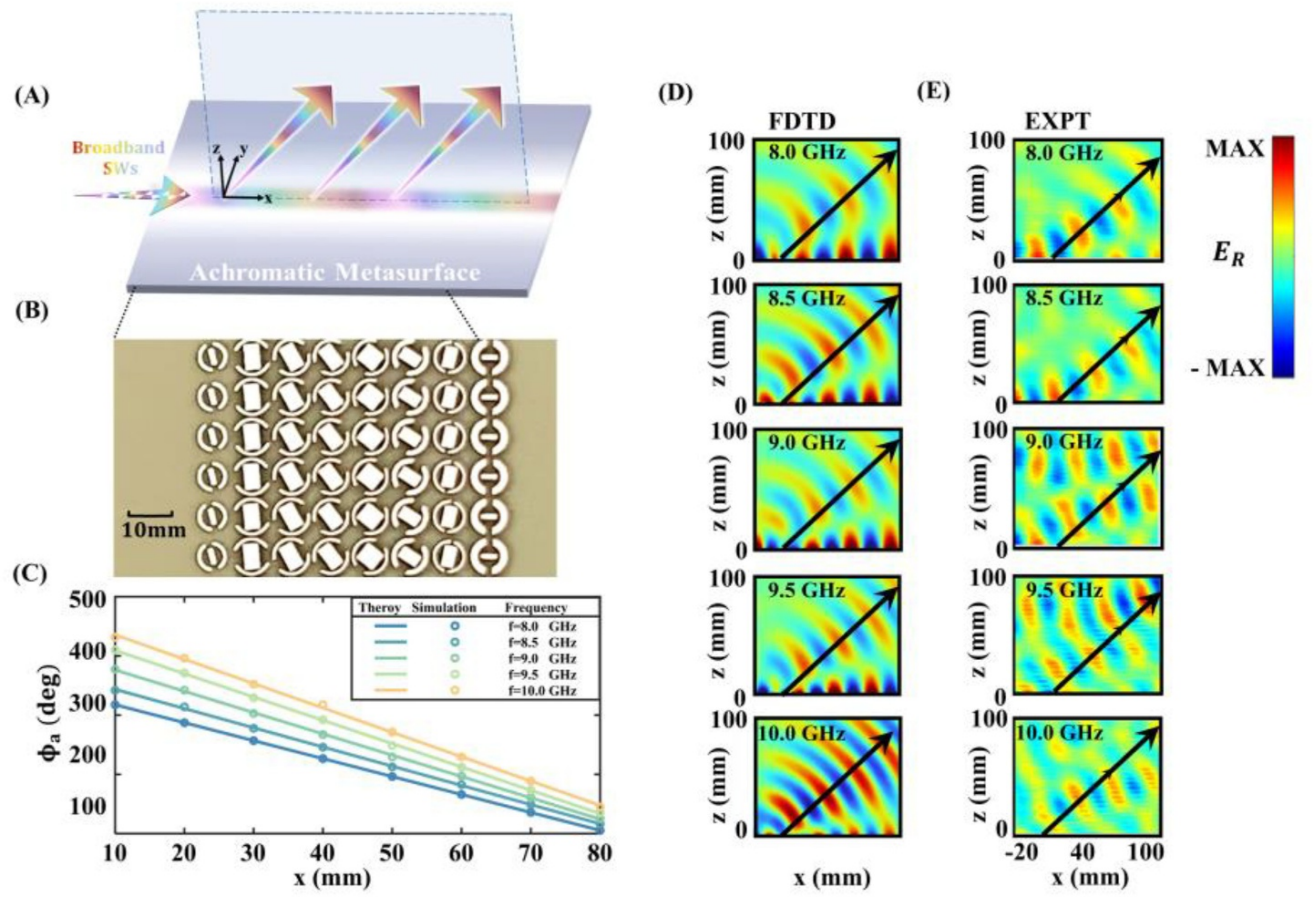
Unidirectional in‐plane radiation of SW by proposed on‐chip achromatic MS. (A) Schematic of achromatic FF radiation of broadband SW based on the on‐chip MS. (B) The fabricated MS sample. (C) Spatial resonant phase profiles of the abnormal radiation mode emitted by the achromatic MS at several representative frequencies. (D) Simulated and (E) measured electric field $E_{R}$ patterns of the radiation beams generated by the SW excited MS at representative frequencies.

Following the aforementioned strategy, we fabricate the sample and adopt the NF mapping technique to verify its achromatic functionality. Figure 3B illustrates the top view of the fabricated MS with the total size of 800 mm × 360 mm (see more detailed structural information in Table S1 of the Supplementary Materials). In our experimental setup, we employ a horn antenna to normally illuminate a meta‐coupler (see Section B of the Supplementary Materials) to excite broadband SWs propagating along + *x* direction on the plasmonic metal. Subsequently, these SWs interact with the achromatic MS and are eventually decoupled to the FF predefined direction. We adopt a monopole antenna to map the electric field distribution of the radiation beam on the XOZ plane at different frequencies. Both antennas are connected to a vector network analyzer (China Electronics Technology Instruments, 3671D). In our measurement, the monopole antenna is controlled by a 3D motorized scanning stage with a constant translation step of 2 mm. We measure different electric field components (including amplitude and phase) by orienting the monopole probe along corresponding directions (*x*, *y* and *z*), respectively. The radiation field exhibiting desired CP can be reconstructed through the analytical recombination of these data. Figure 3E presents experimentally measured $E_{R}$ distributions on the XOZ plane at five representative frequencies (More detailed information of derivation can be found in Section C in Supplementary Materials), showing good agreement with simulation results (see Figure 3D). Here, all simulation results were obtained by Eastwave software, a commercial software based on the finite‐difference‐time‐domain (FDTD) method. In our simulations, an SW beam is generated through a port and propagates from the plasmonic metal to the achromatic metadevice. The scattered waves are dissipated by the surrounding perfectly matched layers and the EM field patterns at various frequencies are collected by a field monitor. Notably, the metadevice successfully transforms impinging SWs to nearly identical in‐plane direction $\left(\theta_{0}=44.6{}^{\circ}\right)$ carrying RCP within the target frequency band of 8–10 GHz.

### 2.3 Out‐Of‐Plane Unidirectional Radiation of SW Across a Broad Band

The proposed strategy is general that can be extended to achieve more sophisticated radiation field manipulations for on‐chip SWs. For example, we demonstrate another achromatic MS capable of decoupling SWs to an out‐of‐plane unidirectional beam (e.g., $\theta_{2}^{0}=48{}^{\circ},\phi_{2}^{0}=22{}^{\circ}$) in 3D space operating across a broader band of 8.8–12.8 GHz (see Figure 4A). To achieve this goal, we introduce a kind of reflective meta‐atoms in a multi‐layer stack configuration. The present design comprises metallic double split‐ring resonators (DSRRs) and a continuous metallic mirror positioned on different XOY planes, which are separated by two F4B dielectric layers, as schematically shown in Figure 5A–C. Notably, the resulting phase dispersion of such design exhibits a steeper slope across the broader frequency band, thanks to the rich coupling effect of multi‐layer configuration.

**FIGURE 4:**
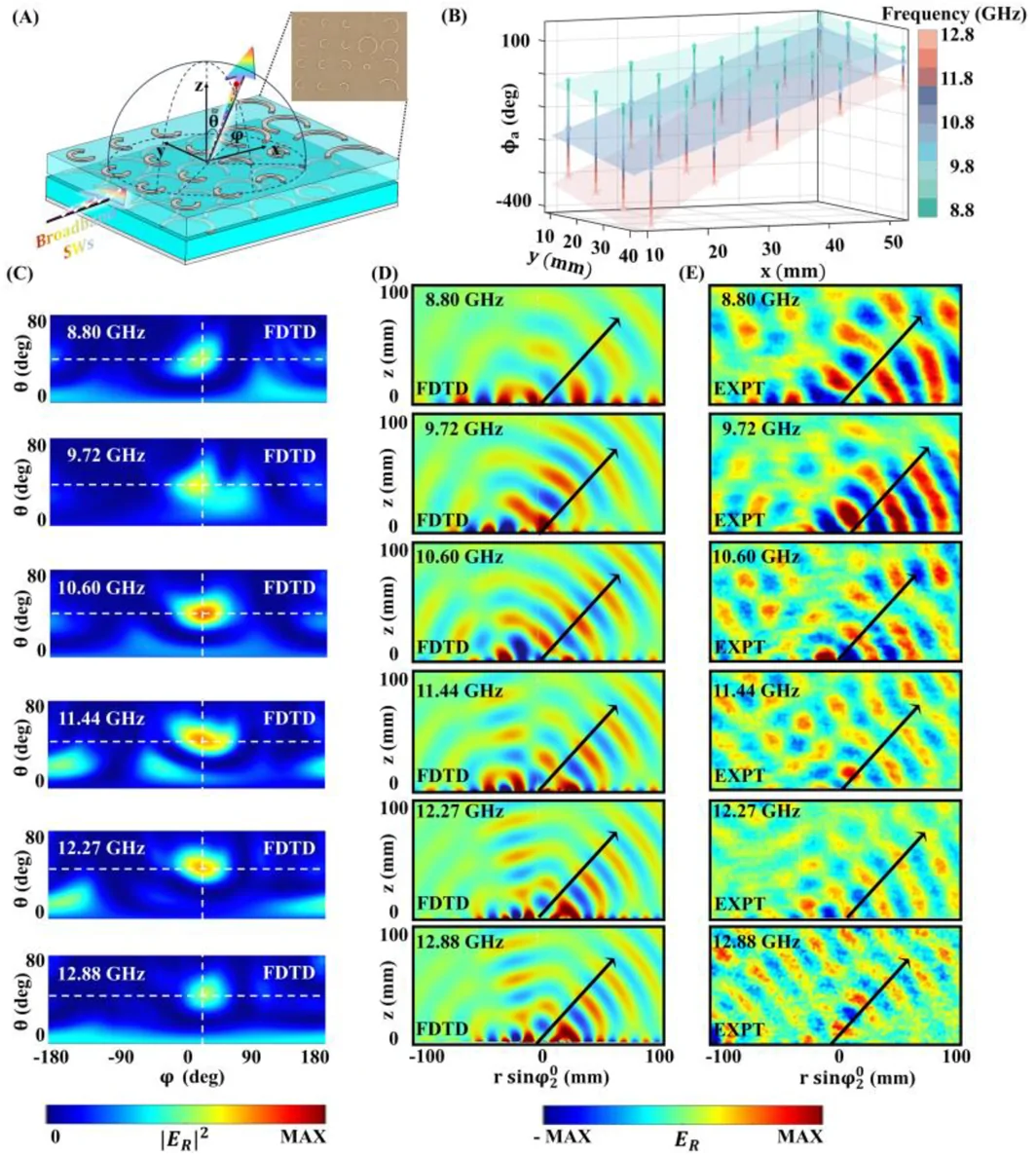
(A) Schematic of unidirectional out‐of‐plane radiation by the second achromatic MS under SW excitation. (B) Spatial reflection phase distributions of the RCP abnormal mode of the MS at three representative frequencies of 8.8 GHz, 10.8 GHz, 12.8 GHz, obtained by ideal model (plane) and practical design (dot). Here, the color denotes the frequency. (C) Simulated angular distributions of radiation beam ${\left|E_{R}\right|}^{2}$ with RCP generated by the SW‐driven on‐chip achromatic MS at six representative frequencies. (D, E) Simulated and measured spatial patterns of $E_{R}$ field on the radiation plane ($\phi_{2}^{0}=22{}^{\circ}$) emitted by the achromatic MS at six representative frequencies.

**FIGURE 5:**
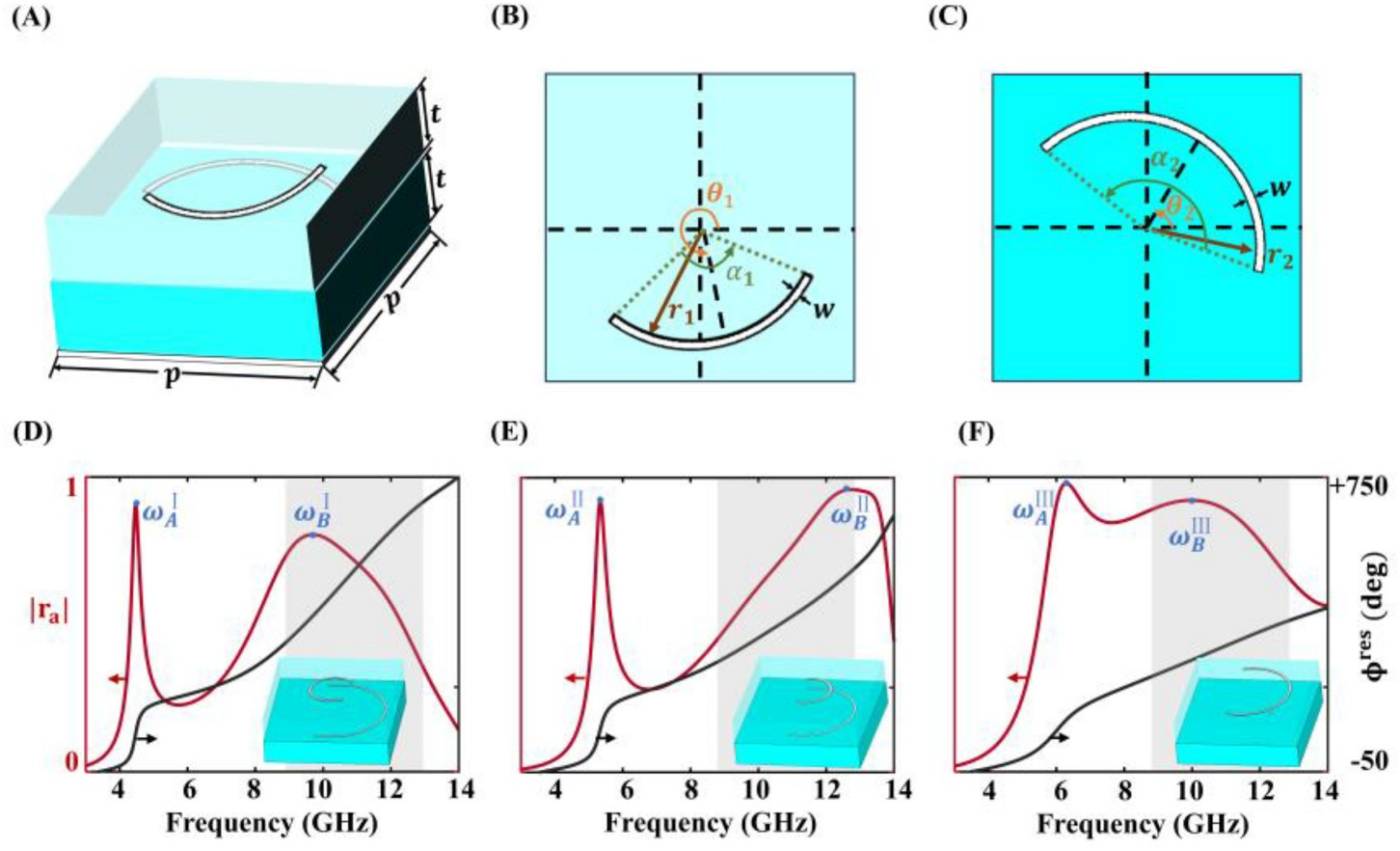
Characterization of representative multilayer meta‐atoms for creating the second achromatic meta‐devices. (A) The meta‐atom is composed of two coupled copper SRRs and a continuous copper film located at different layers that are separated by two 2 mm thick F4B dielectric substrate (*ε*_FR4_ = 3.5 + 0.001*i*). Here, p = 10.4 mm, t = 2 mm. (B, C) Top views of two distinct SRRs located on the upper and lower substrate, respectively. (D, E, F) Calculated spectra of resonant phase and anomalous reflection mode amplitude ($\left|r_{a}\right|$) for the three representative meta‐atoms. These building blocks exhibit quite good linear resonant phase dispersions with different slopes across the target band of 8.8–12.8 GHz. Geometric parameters are listed as follows: w=0.2mm, $r_{1}=1.8\;\text{mm}$, ${\;r}_{2}=4.2\;\text{mm}$, $\alpha_{1}=260{}^{\circ}$, $\alpha_{2}=210{}^{\circ}$, $\theta_{1}=172.5{}^{\circ}$, and $\theta_{2}=0{}^{\circ}$ for meta‐atom I, w=0.2mm, $r_{1}=1.8\;\text{mm}$, ${\;r}_{2}=3.6\;\text{mm}$, $\alpha_{1}=210{}^{\circ}$ , $\alpha_{2}=210{}^{\circ}$, $\theta_{1}=0{}^{\circ}$, and $\theta_{2}=0{}^{\circ}$ for meta‐atom II, $r_{1}=3.6\;\text{mm}$, ${\;r}_{2}=1.8\;\text{mm}$, $\alpha_{1}=210{}^{\circ}$ , $\alpha_{2}=10{}^{\circ}$, $\theta_{1}=0{}^{\circ}$, and $\theta_{2}=0{}^{\circ}$ for meta‐atom III.

We now outline the strategy for designing the meta‐atoms with precisely engineered phase dispersion. As demonstrated by the previous studies [47, 48, 49, 50], such meta‐atoms consisting of DSRRs exhibit rich optical responses originating from the coupling effects that are sensitive to their separation distance [51] and relative twisted angle [52]. Compared with meta‐atoms for achieving in‐plane radiation, such asymmetric DSRRs enable large range of phase modification across the target band relying on the rich hybridization modes. Many parameters can be utilized to modulate electromagnetic responses of such meta‐atoms, including each ring's orientation angle $\theta_{1}$, $\theta_{2}$, spread angle $\alpha_{1}$, $\alpha_{2}$ and radius size $r_{1}$, $r_{2}$, thus offering a database of meta‐atoms with different phase dispersion features across a broader band (8.8–12.8 GHz). For instance, Figure 5D depicts the amplitude and resonant phase spectra of anomalous reflection mode inside the meta‐atom I under the illumination of CP light. We can note that the resonant phase spectra of meta‐atom I shows strong frequency dispersion, thus giving rise to a steep phase slope. For comparison, we present the reflection spectra of meta‐atom II, as shown in Figure 5E. Here, the geometric parameters of the upper and lower SRRs in meta‐atom I and meta‐atom II are nearly identical, except for a relative twisted angles difference of about 180°. We can observe a decreased phase slope in comparison to the spectra in Figure 5D. Moreover, although significantly reducing the size of the lower SRR, we have designed the meta‐atom III, which exhibits a nearly linear resonant phase with a minimum phase slope (see Figure 5F). We note that, two modes appear at significantly separated frequencies (6.2 and 10.3 GHz) with relatively low quality factors, thereby leading to a weak phase dispersion (see more details in Section D of the Supplementary Materials).

With the database of newly developed five‐layer meta‐atoms, we begin to design the achromatic MS that can achieve out‐of‐plane unidirectional radiation of SWs as governed by Equation (6). We need to introduce frequency‐dependent phase gradient along both *x* and *y* directions. Following the strategy as presented in last section, we can integrate all qualified meta‐atoms with precisely tuned orientation angles to construct the second on‐chip achromatic MS, as shown in Figure 4A. More detailed information of this device can be found in Table S2 of Supporting Information. The spatial phase profiles of the designed MS at three representative frequencies (8.8, 10.8, 12.8 GHz) are illustrated by the green, blue, and red dots in Figure 4B, demonstrating excellent agreement with the theoretical design (green, blue, and red planes) derived from Equation (6). We have adopted full wave simulations to obtain the electric field intensity distributions of FF carrying RCP radiated by the achromatic MS under excitation of SW along + *x* direction across various frequencies (see Figure 4C). Via retrieving the angle of the radiation peak in the colormap, we can confirm that the radiation beams appear almost at identical angles of $\theta_{2}^{0}=48{}^{\circ},\phi_{2}^{0}=22{}^{\circ}$ within the broad band of [8.8 GHz, 12.8 GHz].

The fabricated sample, with a total size of 546 mm × 333 mm, contains three main components, the meta‐coupler (see Section E of the Supporting Information S1), the plasmonic metal, and the achromatic SW driven MS (see sample image in Figure 4A). Figure 4E depicts the experimentally measured SW‐PW radiation field pattern with RCP ($E_{R}$) on the radiation plane (determined by the parallel component of $\vec{k}$ vector for achromatic radiation beam and *x* axis, i.e., $\phi_{2}^{0}=22{}^{\circ}$), showing reasonable agreement with the full wave simulation results (See Figure 4D). Here, the retrieval method is also based on the recombination of the different field components $E_{x}$, $E_{y}$, $E_{z}$ (More detailed information of derivation can be found in Section C in Supporting Information S1). We can note that, as broadband SWs flow on the MS, they are gradually decoupled to the predefined direction of $\theta_{2}^{0}=48{}^{\circ}$. Full‐wave simulations demonstrate that the averaged (or highest) efficiencies of two metadevices reach 54.17% and 54.87% (or 70.36% and 86.96%) across the target frequency bands. Here, the efficiency is defined as the ratio between the power carried by the unidirectional radiation beam and that by the incident SW that interacted with the metasurface.

## 3 Conclusions

In summary, we propose a novel scheme for designing the achromatic on‐chip MS that can achieve unidirectional FF radiation under the excitation of broadband NF SWs. Distinct from conventional PW excited achromatic MS, the present on‐chip achromatic metadevices introduce two frequency‐depend phase terms: one is responsible for creating the desired FF wavefront and the other is for compensating the propagation phase of SW propagating on the device. To validate our scheme, we first construct a continuous band achromatic on‐chip MS composed by the MIM typed meta‐atoms encoded with both resonant and PB phases. NF mapping experiments successfully demonstrate its functionality to decouple the SW to predefined in‐plane direction ($\theta_{1}^{0}=44.6{}^{\circ}$, $\phi_{1}^{0}=0{}^{\circ}$) within the frequency range 8–10 GHz, showing good agreement with full wave simulations and theoretical prediction (simulation results in Section F of Supplementary Materials demonstrate that it can indeed work within a broader band). Furthermore, we experimentally demonstrated the second on‐chip MS for unidirectional out‐of‐plane SW radiation ($\theta_{2}^{0}=48{}^{\circ},\phi_{2}^{0}=22{}^{\circ}$), which are constituted by multilayer microstructures exhibiting a larger range of achievable phase slopes within an extended working band (8.8–12.8 GHz). The bandwidth of the achromatic metasurfaces may be further expanded via utilizing more complicated microstructures (e.g., multi‐layered metallic meta‐atoms and high aspect ratio dielectric meta‐atoms) with a larger range of phase retardation. Inspired by numerous literature on both the achromatic metasurfaces and the on‐chip metasurfaces operating at THz or optical band [39, 40, 44, 45], developing the concept of on‐chip achromatic metasurfaces from microwave to high frequency regimes represents a highly promising and methodologically straightforward research direction, provided that several fundamental issues such as high‐precision fabrication and increasing loss are addressed [53, 54]. In comparison with conventional PW‐driven metasurfaces, the proposed on‐chip metadevices demonstrate significant advantages in terms of high integration density and single‐mode operation capability, rendering them particularly promising for next‐generation on‐chip photonic systems. Meanwhile, the current on‐chip devices also face substantial design challenges, primarily due to the inherent spatial non‐uniformity of local field distributions experienced by individual meta‐atoms. This research paves a new paradigm for on‐chip coupler design, with great implications for advancing integrated photonic technologies including planar radar system, optical sensing, photonic circuits, augmented/virtual reality (AR/VR) devices, and so on.

## Author Contributions

**Haodong Li:** conceptualization, writing – original draft, investigation, software, validation, visualization. **Shaojie Ma:** formal analysis, methodology. **Lei Zhou:** resources, supervision, funding acquisition. **Shulin Sun:** conceptualization, methodology, writing – original draft, writing – review and editing, investigation, resources, funding acquisition, supervision, project administration.

## Funding

This work was supported by the National Key Research and Development Program of China (Grant Nos. 2022YFA14047001, 2020YFA0710100, 2023YFA1406900), National Natural Science Foundation of China (Grant Nos. 12374344, 12221004, 12374343, 62192771), Natural Science Foundation of Shanghai (Grant Nos. 23dz2260100, 25TS1415600), The East Talents Program of Shanghai, and Key Projects of “Double First‐Class” Initiative of Fudan University.

## Supporting information


Supporting Information S1

## Data Availability

All data are available in the main text or the Supplementary Materials. Requests should be directed to the corresponding author.
