# Supplementary material for: Unidirectional Far‐Field Radiation of Surface Waves by On‐Chip Achromatic Metasurfaces
Source: Nanophotonics. 2026 Apr 10;15(7):e70064. doi: 10.1002/nap2.70064 (PMC13642102; doi:10.1002/nap2.70064)
Supplement: Supplementary file 1 [file NAP2-15-e70064-s001.docx]

**Supplementary Material**

**Unidirectional far-field radiation of surface waves by on-chip achromatic metasurfaces**

Haodong Li^1,3^, Shaojie Ma^1,3^, Lei Zhou^2,3,*^, Shulin Sun^1,3,*^

*^1^Shanghai Engineering Research Center of Ultra-Precision Optical Manufacturing, Department of Optical Science and Engineering, College of Future Information Technology, Fudan University, Shanghai 200433, China;*

*^2^State Key Laboratory of Surface Physics and Key Laboratory of Micro and Nano Photonic Structures (Ministry of Education), Department of Physics, Fudan University, Shanghai 200433, China*

*^3^Shanghai Key Laboratory of Metasurfaces for Light Manipulation, Shanghai, 200433, China*

Keywords: achromatic metasurfaces; far-field radiation; resonant phase; Pancharatnam–Berry phase; surface wave.

*Corresponding authors:

Shulin Sun: [sls@fudan.edu.cn](mailto:sls@fudan.edu.cn);

Lei Zhou: [phzhou@fudan.edu.cn](mailto:phzhou@fudan.edu.cn).

**Outline**

[A.	Representative meta-atoms of achromatic on-chip metasurface for achieving in-plane unidirectional radiation	3](#_Toc223270165)

[B. Broadband SW meta-coupler for in-plane achromatic radiation	4](#_Toc223270166)

[C. Derivation of unidirectional radiation beam in circular polarization bases	6](#_Toc223270167)

[D. Representative meta-atoms of achromatic metasurface for achieving out-of-plane unidirectional radiation	7](#_Toc223270168)

[E. Broadband SW meta-coupler for out-of-plane achromatic radiation	8](#_Toc223270169)

[F. Unidirectional in-plane radiation of SW by proposed on-chip achromatic MS across a broader frequency band	10](#_Toc223270170)

# A. Representative meta-atoms of achromatic on-chip metasurface for achieving in-plane unidirectional radiation

**Fig. S1.** (A) Surface current (arrow) distributions of two modes appearing at 6.64 GHz and 11.85 GHz inside the meta-atom I under the illuminations of TE and TM polarized plane waves. (B) Magnetic field intensity (color) and surface current (arrow) distributions of three plasmonic resonance modes (appearing at 7.40 GHz, 9.03 GHz and 14.66 GHz) excited inside the meta-atom II under the illuminations of different linearly polarized plane waves. (C, D) Simulated spectra of resonant phase and abnormal reflection mode amplitude (${|r}_{a}|$) for the two representative meta-atoms obtained. It is noted that these building blocks exhibit quite high-efficiency and good linear resonant phase dispersion with different slopes across the target band of 8-10 GHz.

# B. Broadband SW meta-coupler for in-plane achromatic radiation

**Fig. S2.** Sample picture (A) and schematic illustration (B) of the SW meta-coupler designed for the first achromatic on-chip metasurface (MS) in the main text. (C, D) Schematic of a unit cell consisting of a curved H-shaped metallic microstructure and a continuous metallic mirror separated by a dielectric FR4 spacer ($\varepsilon_{FR4}=4.3+0.005i$). Here, the following parameters are constants: $p$=10 mm, $\alpha$=${116}^{^{\circ}}$, $l$=8.3 mm, $w$=0.1 mm, $t$=2 mm. The meta-coupler is composed by a series of PB meta-atoms with indentical geometries but a constant rotation angle step of ${58}^{^{\circ}}$. (E) Simulated $\left| r_{a} \right|, \phi_{uu}, \phi_{vv}$ of the PB meta-atoms illuminated by the normally incident waves within 8-10 GHz**.**

**Fig. S3.** Simulated $E_{x}$ field pattern in the XOZ plane (y=0 mm) generated by the proposed SW meta-coupler under the illumination of RCP plane waves. We can note that SW beam can be excited the plasmonic metal across the broad band of 8-10 GHz.

# C. Derivation of unidirectional radiation beam in circular polarization bases

　 In this section, we introduce the derivation of the radiation far-field carrying specific circular polarization. The wavevector of unidirectional radiation beam generated by the proposed SW excited achromatic MS can be described as:

$\vec{k}=k_{x}\hat{e}_{x}+k_{y}\hat{e}_{y}+k_{z}\hat{e}_{z}$	(S1)

where $k_{x}=k_{0}sin\theta cos\varphi$, $k_{y}=k_{0}sin\theta sin\varphi$, $k_{z}=k_{0}cos\theta$, and $\theta$, $\varphi$ denote radiation angle of the unidirectional radiation beam.

We select two local unit vectors $\hat{e}_{x}^{'}$ and $\hat{e}_{y}^{'}$ as local reference axes and derive their analytical expressions of radiation beam with desired polarization. Subsequently, the unit vectors for the radiation beam exhibiting circular polarization are defined as $\hat{e}_{\pm}=\hat{e}_{x}^{'}\pm i\hat{e}_{y}^{'}$. Given that both $\hat{e}_{x}^{'}$ and $\hat{e}_{y}^{'}$ lie in the plane perpendicular to the propagation direction (i.e.,$\vec{k}$) of radiation beam, we can obtain the following equation:

$$\hat{e}_{y}^{'}= \frac{\vec{k}}{\left| \vec{k} \right|}\times\hat{e}_{x}^{'}=\left[ \begin{matrix} \hat{e}_{x} & \hat{e}_{y} & \hat{e}_{z} \\ sin\theta cos\varphi& sin\theta sin\varphi& cos\theta\\ sin\varphi& -cos\varphi& 0 \end{matrix} \right]$$

$=(cos\theta cos\varphi\hat{e}_{x}+cos\theta sin\varphi\hat{e}_{y}-sin\theta\hat{e}_{z})$	(S2)

We then obtain the unit vectors of CP for the radiation fields:

$\hat{e}_{\pm}=\hat{e}_{x}^{'}\pm i\hat{e}_{y}^{'}=(sin\varphi\pm icos\theta cos\varphi)\hat{e}_{x}-(cos\varphi\mp icos\theta sin\varphi)\hat{e}_{y}\mp isin\theta\hat{e}_{z}$ (S3)

Finally, we can derive the electric field distribution carrying CP based on the three components in the laboratory coordinate system:

$E_{\pm}=\hat{e}_{\pm}\cdot\overset{⃑}{E}=\left( sin\varphi\pm icos\theta cos\varphi\right)E_{x}-\left( cos\varphi\mp icos\theta sin\varphi\right)E_{y}\mp isin\theta E_{z}$	(S4)

# D. Representative meta-atoms of achromatic metasurface for achieving out-of-plane unidirectional radiation

**Fig. S4.** (A) Surface current (arrow) distributions of two modes (appearing at 4.46 GHz and 9.70 GHz) excited inside the meta-atom I under the illuminations of TE and TM polarized plane waves. (B) Surface current (arrow) distributions of two modes (appearing at 5.33 GHz and 12.60 GHz) excited inside the meta-atom II under the illuminations of different linearly polarized plane waves. (C) Surface current (arrow) distributions of two modes (appearing at 6.20 GHz and 9.97 GHz) excited inside the meta-atom III under the illuminations of different linearly polarized plane waves. (D, E, F) Simulated spectra of resonant phase and abnormal reflection mode amplitude (${|r}_{a}|$) for the three representative meta-atoms. It is noted that these building blocks exhibit quite high-efficiency and good linear resonant phase dispersion with different slopes across the target band of 8.8-12.8 GHz.

# E. Broadband SW meta-coupler for out-of-plane achromatic radiation

**Fig. S5.** Sample picture (A) and schematic illustration (B) of the SW meta-coupler designed for the second on-chip achromatic MS in the main text. (C, D) Schematic of one representative meta-atom consisting of a H-like metallic microstructure and a contineous metallic mirror separated by a dielectric F4B spacer. One super cell of the meta-coupler is composed of 4 sub-cells with the varying arm length of 2.20 mm, 3.05 mm, 3.55 mm, 4.30 mm, respectively. The other parameters are fixed: $p$=4.8 mm, $w$=0.3 mm, $g$=0.3 mm, $t$=4 mm. (E) Simulated spatial distributions of reflection phase $\phi_{xx}$ inside the meta-coupler illuminated by the normally incident x-polarized propagation waves at the frequency of 10.8 GHz.

**Fig. S6.** Simulated $E_{x}$ field pattern in the XOZ plane (y=0 mm) generated by the proposed SW meta-coupler under the illumination of $x$-polarized plane waves within the band of 8.8-12.8 GHz.

# F. Unidirectional in-plane radiation of SW by proposed on-chip achromatic MS across a broader frequency band

**Fig. S7.** Simulated $E_{R}$ field intensity of the radiation beam on the XOZ plane (y=0 mm) generated by the first achromatic metasurface across a broader frequency range of [7.75 GHz, 10.5 GHz]. It is noted that the achromatic in-plane beam radiation effect can maintain well across the whole band.

**Table S1**: Structural parameters of adopted meta-atoms for creating achromatic metasurface to achieve in-plane unidirectional radiation.

| $x_{i}$ | r (mm) | L (mm) | w (mm) | g (mm) | c (mm) | Rotation angle (${}^{^{\circ}}$) |
| --- | --- | --- | --- | --- | --- | --- |
| $x_{1}$ | 3.00 | 3.86 | 1.04 | 0.5 | 2.0 | -165.25 |
| $x_{2}$ | 4.15 | 3.65 | 6.32 | 0.5 | 4.0 | -84.85 |
| $x_{3}$ | 4.15 | 3.65 | 6.32 | 0.5 | 3.0 | -60.95 |
| $x_{4}$ | 4.00 | 3.50 | 6.06 | 0.5 | 3.0 | -53.05 |
| $x_{5}$ | 4.00 | 4.77 | 5.12 | 0.5 | 3.0 | -39.00 |
| $x_{6}$ | 3.50 | 3 | 5.20 | 1.0 | 3.0 | -32.65 |
| $x_{7}$ | 3.50 | 5.20 | 3.00 | 0.5 | 1.0 | -12.35 |
| $x_{8}$ | 3.00 | 1.04 | 3.06 | 1.5 | 2.0 | -1.40 |

**Table S2**: Structural parameters of adopted meta-atoms for creating achromatic metasurface to achieve out-of-plane unidirectional radiation.

| ($x_{i},y_{j}$) | $r_{1}$ (mm) | $\alpha_{1}$ (${}^{^{\circ}}$) | $\theta_{1}$ (${}^{^{\circ}}$) | $r_{2}$ (mm) | $\alpha_{2}$ (${}^{^{\circ}}$) | $\theta_{2}$ (${}^{^{\circ}}$) | Rotation angle (${}^{^{\circ}}$) |
| --- | --- | --- | --- | --- | --- | --- | --- |
| $(x_{1},y_{1}$) | 1.8 | 260.0 | 172.5 | 4.2 | 210.0 | 0.0 | -57.7 |
| $(x_{1},y_{2}$) | 1.8 | 260.0 | 172.5 | 4.2 | 210.0 | 0.0 | -67.4 |
| $(x_{1},y_{3}$) | 1.8 | 260.0 | 170.0 | 4.2 | 210.0 | 0.0 | -77.8 |
| $(x_{1},y_{4}$) | 1.8 | 260.0 | 167.5 | 4.2 | 210.0 | 0.0 | -88.1 |
| $(x_{2},y_{1}$) | 1.8 | 210.0 | 155.0 | 4.2 | 210.0 | 0.0 | -39.6 |
| $(x_{2},y_{2}$) | 1.8 | 210.0 | 150.0 | 4.2 | 210.0 | 0.0 | -50.3 |
| $(x_{2},y_{3}$) | 1.8 | 210.0 | 145.0 | 4.2 | 210.0 | 0.0 | -60.7 |
| $(x_{2},y_{4}$) | 1.8 | 210.0 | 140.0 | 4.2 | 210.0 | 0.0 | -70.9 |
| $(x_{3},y_{1}$) | 1.8 | 210.0 | 0.0 | 3.6 | 210.0 | 0.0 | -34.9 |
| $(x_{3},y_{2}$) | 2.4 | 160.0 | 0.0 | 3.6 | 210.0 | 0.0 | -42.5 |
| $(x_{3},y_{3}$) | 1.8 | 210.0 | 170.0 | 4.2 | 210.0 | 0.0 | -34.4 |
| $(x_{3},y_{4}$) | 1.8 | 210.0 | 170.0 | 4.2 | 210.0 | 0.0 | -44.1 |
| $(x_{4},y_{1}$) | 1.8 | 10.0 | 0.0 | 3 | 310.0 | 0.0 | 23.1 |
| $(x_{4},y_{2}$) | 1.2 | 310.0 | 0.0 | 4.8 | 310.0 | 0.0 | 13.4 |
| $(x_{4},y_{3}$) | 4.2 | 310.0 | 0.0 | 2.4 | 310.0 | 0.0 | -9.0 |
| $(x_{4},y_{4}$) | 4.8 | 10.0 | 0.0 | 3.6 | 310.0 | 0.0 | -52.1 |
| $(x_{5},y_{1}$) | 4.8 | 160.0 | 0.0 | 4.8 | 110.0 | 0.0 | -24.2 |
| $(x_{5},y_{2}$) | 4.2 | 160.0 | 0.0 | 2.4 | 210.0 | 0.0 | -39.2 |
| $(x_{5},y_{3}$) | 3.6 | 260.0 | 0.0 | 1.8 | 60.0 | 0.0 | -8.3 |
| $(x_{5},y_{4}$) | 3.6 | 210.0 | 0.0 | 1.8 | 10.0 | 0.0 | -46.6 |
